# Effects of Ankaferd Hemostat on Helicobacter pylori strains and antibiotic resistance

**DOI:** 10.3906/sag-1807-206

**Published:** 2019-02-11

**Authors:** Rafiye ÇİFTÇİLER, Ahmet KOLUMAN, İbrahim C. HAZNEDAROĞLU, Nejat AKAR

**Affiliations:** 1 Department of Adult Hematology, Faculty of Medicine, Hacettepe University, Ankara Turkey; 2 Department of Biomedical Engineering, Faculty of Technology, Pamukkale University, Denizli Turkey; 3 Department of Pediatric Hematology, Faculty of Medicine, TOBB-ETÜ Hospital, Ankara Turkey

**Keywords:** Ankaferd blood stopper, gastrointestinal bleeding, *Helicobacter pylori*

## Abstract

**Background/aim:**

Ankaferd hemostat (ABS; Ankaferd blood stopper, İstanbul, Turkey) is a folkloric medicinal plant extract. The aim of this study was to determine the effect of Ankaferd hemostat (ABS) on the fate of *Helicobacter pylori* strains. The study also aims to determine alterations in the antimicrobial resistance of three different *H. pylori* strains in response to ABS exposure.

**Materials and methods:**

*H. pylori* Strain 1 was obtained from the culture collection ATCC 43504 and passaged three times for viability. Strain 2 was isolated from a gastric ulcer patient and Strain 3 was isolated from a gastritis patient. 1% of ABS was added to all of the strains and antimicrobial susceptibility was observed on 30 and 60 min after application.

**Results:**

The efficacy of ABS solutions in achieving significant logarithmic reduction in foodborne pathogens of *H. pylori* was observed in this study. This study showed that ABS has antibacterial (Anti-*H. pylori*) effects.

**Conclusion:**

Our present study indicated, for the first time, that ABS could act against *H. pylori*. ABS is clinically used for the management of GI bleeding due to benign and malignant GI lesions. Thus, the possible anti-*H. pylori* effect of ABS shall expand the therapeutic spectrum of the drug in GI lesions in relation to *H. pylori* infection such as peptic ulser disease (PUD) and lymphoid tissue (MALT) lymphomagenesis**.**

## 1. Introduction

Ankaferd hemostat (ABS; Ankaferd blood stopper, İstanbul, Turkey) is a folkloric medicinal plant extract. ABS has been conventionally used in Anatolia as a hemostatic agent for centuries (1). ABS contains a standardized combination of the plants *Glycyrrhiza glabra*, *Thymus vulgaris*, *Alpinia ofﬁcinarum*, *Vitis vinifera*, and *Urtica dioica*. All of these plants have effects on the endothelium, the cellular components of blood, the development of new blood vessels, cell proliferation, and cell mediators (2,3). The hemostatic effect of ABS depends upon the quick promotion of a protein network, particularly fibrinogen gamma, in relation to the erythrocyte aggregation (1).

In addition to hemostatic functions, antiinflammatory (4), antiinfective (5), antifungal (6), and antioxidative (4) effects have been demonstrated for ABS. *Thymus vulgaris* has bacteriostatic activity for gram-positive and gram-negative bacteria (7–10). Likewise, *Glycyrrhiza glabra*, *Vitis vinifera*, and *Alpinia officinarum* have been shown to be antibacterial agents (11,12). *Urtica dioica* also has significant antibacterial activity against *Streptococcus pyogenes*, *Staphylococcus aureus*, and *Staphylococcus epidermidis *(12).

ABS is currently licensed for numerous bleeding lesions of GIS pathologies, such as peptic ulcers (13), fundal varices (14), dieulafoy lesions (15,16), radiation colitis (17), rectal ulcers (18) and nonvariceal upper gastrointestinal bleeding (19). Most of the bleeding lesions were controlled with ABS in all patient groups (20–23). Furthermore, ABS is a potent hemostatic drug for controlling malignant gastrointestinal (GI) tumors. It has successful antineoplastic effects on colon cancer (as represented by the in vitro effects on CaCo-2 cells) (24). It significantly decreases tumor microvessel density (25). Almost 70% of gastric ulcer and 85% to 95% of duodenal ulcer patients have coexisting* H. pylori* infections (26,27).  It is well recognized that *H. pylori* eradication therapy can reduce the repetition of peptic ulcer. Several different studies support the important role played by *H. pylori* in mucosa-associated lymphoid tissue (MALT) lymphomagenesis. Chronic infection with *H. pylori* is significantly related with the induction of gastric lymphoid follicles, representing the proposed first step in MALT lymphomagenesis of lymphoid enlargement (28). 

This study was carried out to determine the effects of ABS on the fate of *H. pylori* strains. Our study also aimed to elucidate alterations in the antimicrobial resistance of three different *H. pylori* strains. We also intended to determine alcohol levels in ABS samples in order to elucidate the impact of ABS over gastric acidity and possible effects on gastric mucosa. Informed consent was obtained from blood donors for the procedure for this study.

## 2. Materials and methods

### 2.1. Strains

**Figure 1:**
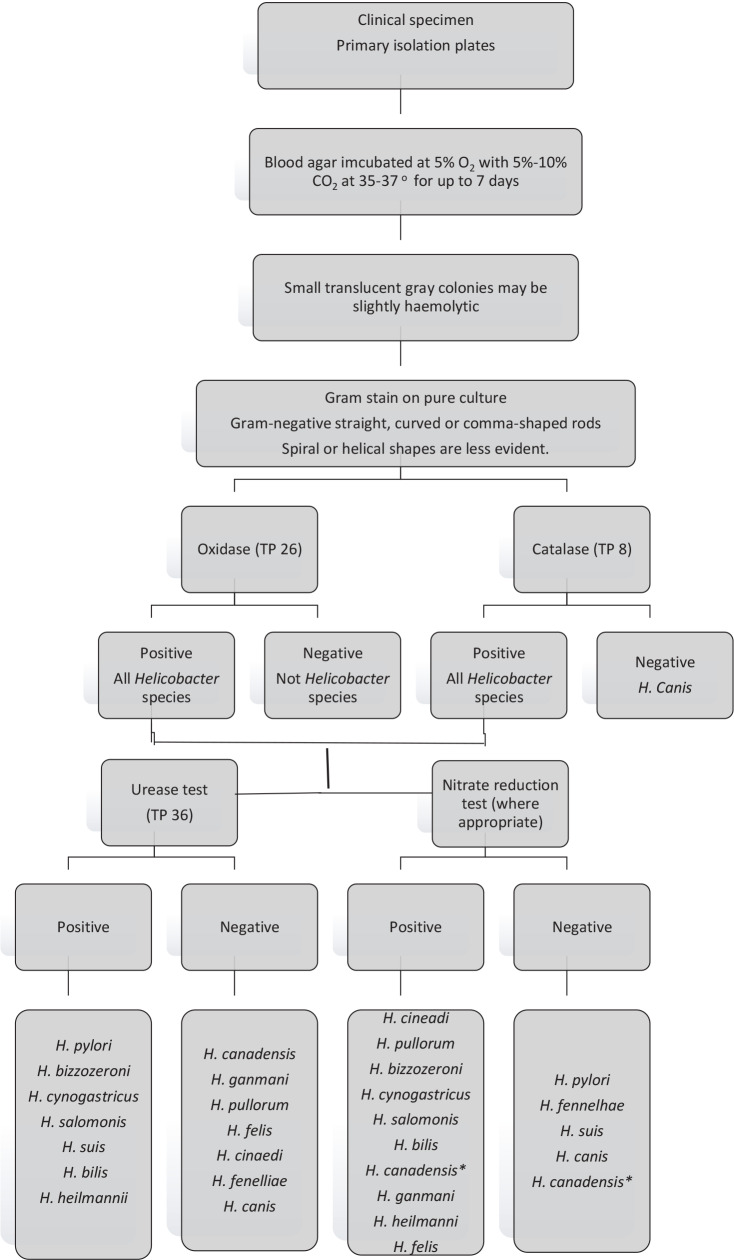
Biochemical identification of Helicobacter spp.

Strain 1 was obtained from a culture collection ATCC 43504 and passaged three times for viability. Strain 2 was isolated from a gastric ulcer patient and Strain 3 was isolated from a gastritis patient. Wild strains were identified using biochemical scheme summarized in Figure 1 and 16s rRNA sequencing was held for wild strains for molecular confirmation of biochemical results. 

**Table 1:** The effects of ABS on the studied bacteria

Consantration(%)	Time (second)									
	5	60	90	120	180	300	600	900	1800	3600
0.5	6.89 ± 0.01^Aw^	6.89 ± 0.02^Aw^	6.86 ± 0.03^Aw^	6.78 ± 0.08^Aw^	6.40 ± 0.18^Aw^	5.98 ± 0.1^BCw^	5.46 ± 0.33^CDw^	5.11 ± 0.2^DEw^	4.71 ± 0.29^EFw^	4.23 ± 0.3Fw
1	6.88 ± 0.02^Aw^	6.80 ± 0.04^Awx^	6.54 ± 0.27^Awx^	6.27 ± 0.24^BCwx^	6.04 ± 0.1^Cw^	5.90 ± 0.12^CDw^	5.56 ± 0.09^Dw^	4.90 ± 0.03^Ew^	4.29 ± 0.23^Fw^	3.98 ± 0.03^Fw^
1.5	6.86 ± 0.01^Aw^	6.73 ± 0.11^Awx^	6.45 ± 0.32^Awx^	6.12 ± 0.17^BCwx^	5.92 ± 0.09^Bw^	5.60 ± 0.25^Cw^	4.86 ± 0.2^Dwx^	4.02 ± 0.07^Ex^	3.79 ± 0.18^EFw^	3.25 ± 0.27^Fx^
2	6.88 ± 0.02^Aw^	6.52 ± 0.17^Awxy^	6.27 ± 0.37^Awx^	5.59 ± 0.49^BCxy^	5.00 ± 0.35^CDx^	4.59 ± 0.28^DEx^	4.04 ± 0.09^EFxy^	3.64 ± 0.38^Fxy^	3.17 ± 0.38^FGwx^	2.34 ± 0.29^Gy^
2.5	6.86 ± 0.03^Aw^	6.39 ± 0.27^ABxy^	6.12 ± 0.25^ABx^	5.45 ± 0.38^ABCy^	4.69 ± 0.38^BDx^	4.15 ± 0.35^CDExy^0	3.45 ± 0.68^DEy^	2.96 ± 0.6^EFy^	1.69 ± 1.49^FGxy^	0.00 ± 0.0^Gz^
3	6.78 ± 0.15^Aw^	6.18 ± 0.21^ABy^	5.96 ± 0.17^Bx^	4.99 ± 0.52^Cy^	4.38 ± 0.46^Cx^	3.52 ± 0.32^Dy^	2.50 ± 0.22^Ez^	0.00 ± 0.0^Fz^	0.00 ± 0.0^Fy^	0.00 ± 0.0^Fz^

A-G: Means in the same line with different superscripts are statistically different (P < 0.05). w-z: Means in the same column with different superscripts are statistically different (P < 0.05).

### 2.2. Biochemical identification of Helicobacter spp. 

Identification of different *Helicobacter *spp*.* was done using biochemical tests. (Anonymous. Identification of Helicobacter species. UK Standards for Microbiology Investigations. Bacteriology – Identification | ID 26, Issue no: 3, Issue date: 03.07.15, Page: 2 of 27)

Strains were streaked on Colobia Agar Base (CM0331, Oxoid, England) supplemented with DENT (Vancomycin, Trimethoprim, Cefsulodin, Amphotericin B) (SR0147, Oxoid, England). All strains were incubated at 37 °C under microaerophilic conditions (CampyGen, CN025, Oxoid, England) for 5–10 days.

### 2.3. Response of H. pylori strains to ABS 

All strains were collected in a tube using a sterile swab and washed using sterile saline (0.85%) three times. Each strain was mixed to form a final cocktail of *H. pylori* and were grouped to determine response to 0.5%, 1%, 1.5%, 2%, 2.5%, and 3% of ABS at 5, 60, 90, 120 s and 3, 5, 10, 15, 30, and, 60 min. This study was repeated three times. 

### 2.4. Changes in antibiotic susceptibility of H. pylori strains after ABS applications

One percent ABS was added to all strains and antimicrobial susceptibility was held at 30 and 60 min of application. Clarithromycin (CT 1623, Oxoid, England), Metronidazole (CT 0067, Oxoid, England), Tetracycline (CT 0054, Oxoid, England), and Amoxicillin (CT0161, Oxoid, England) discs were used to determine susceptibility changes. The following reference strains are used: *Staphylococcus aureus* ATCC® 25923, *Escherichia coli* ATCC® 25922, *Pseudomonas aeruginosa* ATCC® 27853, *Haemophilus influenza* ATCC® 49247, *Neisseria gonorrhoeae* ATCC® 49226, *Streptococcus pneumoniae *ATCC® 49619, *Escherichia coli* ATCC® 35218 and *H. pylori *ATCC 43504. Results were evaluated using the EUCAST 2016 breaking points. Informed consent was not taken for the reason that people are not included at this step.

## 3. Results

**Figure 2:**
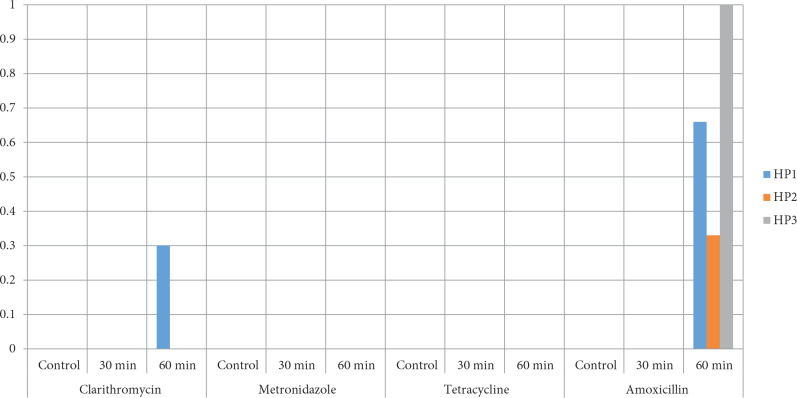
The changes in antibiotic susceptibility after ABS application for determined periods.

The results indicating the effects of ABS on the studied bacteria are depicted in u290b. The relative efficacy of ABS solutions to achieve significant logarithmic reduction in foodborne pathogens *H. pylori. *The distribution of antibiotic susceptibility application for determined periods is depicted in Figure 2.

**Figure 3:**
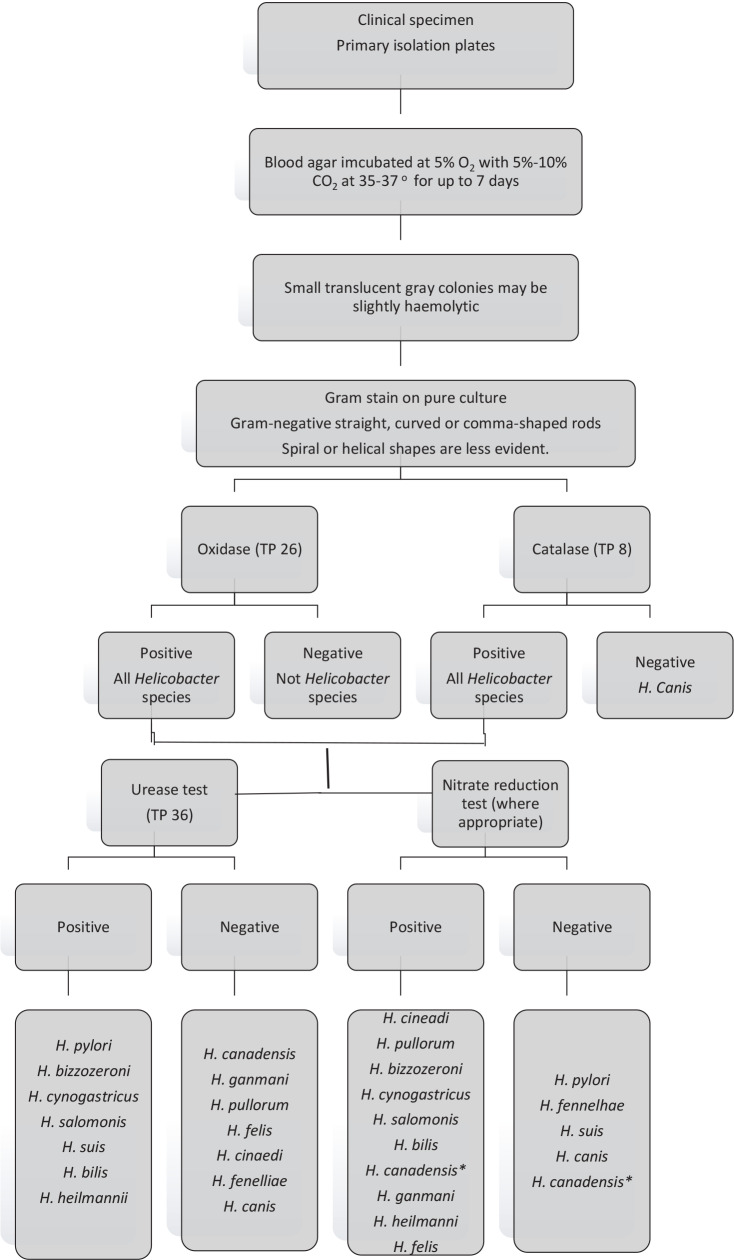
Peak table-channel.

The alcohol ingredient studies revealed a small ethanol peak which was thought to be found by fermentation of grape seeds. Additionally, ethyl acetate was found in the ABS. The amounts of these ingredients were less those found in kefir. The alcohol ingredient studies are depicted in Figure 3 and Table 2. Technical appendix, statistical code, and dataset available from the corresponding author at rafiyesarigul@gmail.com

**Table 2:** The alcohol ingredient study.

Peak table-channel						
Peak	Time	Area	Height	Concentration	Units	Name
1	4.833	239	92	0.000	g/hL	ethyl acetate
2	6.44	21041	5504	0.000	g/hL	ethanol
Total		21280	5596			

## 4. Discussion

In this study, the efficacy of ABS was demonstrated in the antibiotic resistant *H. pylori* strain for the first time. There are no published studies in the literature investigating whether ABS was effective on antibiotic-resistant* H. pylori *strain. With this study, we can hypothesize that ABS in the stomach increases oxygenation and forms the basis for *H. pylori* eradication.

Our findings, in this study, further supported previous research findings that ABS has antibacterial effect (5, 29). Each of herbaceous plants in ABS has known effects on blood cells, endothelium, angiogenesis, cell proliferation, and other physiological mediators (1). *Thymus vulgaris* has antioxidative properties and antimicrobial activity (3). Recent reports have shown that *Glycyrrhiza glabra* has antimicrobial, antioxidant, antifungal as well as antiinflammatory effects (30). *Alpinia officinarum* has antioxidant and antimicrobial activity. (31). *Vitis vinifera* seed extract is related with a large spectrum of pharmacological effects including antioxidative, antiinflammatory and antimicrobial effects (32).  Likewise, *Urtica dioica* has been reported as an antimicrobial agent in pharmaceutical and food industry (33). Anti-*H. pylori* effect of ABS could be related with the protein library of the drug. Functional proteomic analyses were previously performed by Demiralp et al. regarding proteomics of ABS and its effect (34).

The antimicrobial activity of ABS has been demonstrated against many microorganisms (29). Some studies have shown that ABS is highly effective against several gram-negative and gram-positive bacteria including frequent foodborne microorganisms (35). ABS suppresses the development of several common origins of nosocomial infections, including methicillin-resistant *Staphylococcus aureus*, *vancomycin-resistant enterococci*, and *imipenem-resistant Acinetobacter* isolates (5). Several recent reports demonstrated in vitro antibacterial activity of ABS against other multiantibiotic-resistant bacteria, such as *E. coli*, *Enterococcus *spp*.*, *Pseudomonas *spp*.*, and *Klebsiella *spp*.*, also fungi including *Aspergillus *spp*.,* *Candida albicans*, and *Mucor spp.* (36). Koluman et al. showed that ABS inhibits the in vivo growth of gram-positive and gram-negative bacteria (37). Exposure to ABS may support enhanced oxygenation through erythrocyte aggregation (29,36,38). This study also showed that Ankaferd has anti-*H. pylori* effects.

Wound healing, hemostasis, and infection are pathobiologically connected to each other (39). Antithrombin and prohemostatic activities of ABS are related to fibrinogen gamma chain and prothrombin by functional proteomic analyses. (40). Gastric ulcers are in general caused by *H. pylori* and the chronic use of antiinflammatory medications. The antioxidant components of ABS modulate the cellular proliferation and vascular dynamics as well as the hemostatic hemodynamic activity (41). ABS might be beneficial by protecting the gastric mucosa from oxidative injury or by accelerating the healing of gastric ulcers (42). Another study showed that ABS was associated with significantly healed gastric mucosal structure. This might be due to the antioxidant and gastroprotective effects of ABS (43). ABS can improve the wound-healing process by providing inhibition of extra cellular matrix-degrading enzymes during wound repair. ABS enhanced the stimulated migration of 3T3 fibroblasts to an artificial wounded area. The antibiofilm activity of ABS against oral *streptococci* was revealed with in vitro analysis by Boran et al. (44). Therefore, oral administration of ABS could not only treat GI hemorrhage but also ongoing infections and wound-related pathologies as well (43–45).

Herbal medicines could increase the abundance of many bacteria known to support human health status, involving *Bifidobacterium *spp*., Lactobacillus *spp*.*, and *Bacteroides *spp. (46). Peterson et al. suggested that herbal medicine may induce blooms of butyrate- and propionate-producing species. *Ulmus rubra* significantly increased the relative abundance of butyrate-producing bacteria, whereas *Glycyrrhiza glabra* induced the largest increase in propionate-producing species (46). *Glycyrrhiza glabra* is an essential component of ABS (38). Zhou et al. indicated that *Vitis vinifera* have potential prebiotic effects on modulating the gut microbiota composition and generating SCFAs that contribute to the improvements of host health (47). Mandalari et al. also showed that the supplementation with *Vitis vinifera* tended to promote the proliferation of *Bifidobacterium *spp. (48). *Vitis vinifera* is also present in the standardized extract of ABS (38). *Bifidobacterium bifidum *BF-1 suppresses *H. pylori*-induced genes in human epithelial cells. The preincubation with BF 1 strain, a probiotic strain *H. pylori*-associated gastritis, suppresses induction of IL-8 by the pathogen (49). Thus, ABS might contribute to the treatment of *H. pylori* both directly and indirectly via improving biological microbiota such as increasing *Bifidobacterium* spp. of ABS. Future experimental in vivo and/or clinical studies are needed to demonstrate whether ABS had any influence on the growth of the microbioata including *Bifidobacteria.*

There are pathobiological associations among *H. pylori* and lymphoma development (28). Preliminary evidence that ABS has antilymphoma effects in vitro (50) and current findings that ABS has anti-*H. pylori *effects. Oral ABS administration may alter *H. pylori* growth for the prevention of neoplastic lymphoid mucosal tissue proliferation; a hypothesis that should be tested in future experimental approaches. The involvement of *H. pylori* in MALT lymphoma is well established and based on the epidemiological, pathological, clinical, and bacteriological evidence (28). Akalın et al. proposed that ABS can cause apoptosis in the lymphoid neoplastic cells since there was a high content of human IL-4 in ABS solution. They showed that ABS-treated B-chronic lymphocytic cells (B-CLL) (at doses of 0.5, 1, and 2 µg/mL) ceased to inflate and more than 50% of tumor cells died compared to 0.1 and 0.25 µg/mL doses. Moreover, the transformation of B-CLL cells to the blastic aggressive lymphoid forms was prevented by the addition of ABS to the culture medium (50). As presented in our current study, the anti-*H. pylori *effect of ABS may be linked to the previously demonstrated anti-lymphoma effects of the drug. ABS shall be considered as beneficial in the associated lymphoid malignancies. Future experimental in vivo and/or clinical studies are needed to shed further light on the interrelationship between neoplastic GI lymphoid mucosal tissue and antineoplastic within the context of antiproliferative actions. In this study, we showed the efficacy of ABS solutions, including the logarithmic reduction *H. pylori *and foodborne pathogens.

There is a growing body of argument for the accomplished use of ABS in different states of GI bleeding. In an observational study of “intention-to-treat” analysis by Ozaslan et al. (51), ﬁve patients with bleeding peptic ulcers were treated with ABS as the primary hemostatic agent. Similarly in a case report by Purnak et al., a successful control of bleeding was reported in a peptic ulcer patient (13). ABS was selected due to the impotence and difﬁculties of the traditional antihemorrhagic protection agents (19). Endoscopic topical administration of ABS for neoplastic gastrointestinal bleeding was also effectively shown in many studies (52,53). The case series by Kurt et al. showed that local administration of ABS to patients with neoplastic upper GI bleeding provided hemorrhage control and no complications were seen following the procedure (53). Thanks to the mechanical hemostasis obtained by ABS, Turhan et al. (25) reported that ABS reduces tumor vascularization in gastrointestinal carcinoma bleeds. In another report, local ABS administration was applied in two patients with GI bleeds as a result of rectal and gastric neoplasm. Local ABS administration to the lesion was shown to control the bleeding completely. Based on these results, the authors suggested that a secondary and more permanent mechanism of hemostasis over the initial protein network might have been started by ABS. In addition, there is no alcohol in ABS. Therefore, there is no risk of any gastric mucosal damage due to this reason.

In conclusion, the most striking result of our investigation is the documentation of a remarkable antimicrobial activity of ABS against three different strains of *H. pylori.* The pleiotropic effects of ABS on the blood cells, vascular endothelium angiogenesis, cellular proliferation, vascular dynamics, and cellular mediators should be researched to determine its capacity role in wound-healing (2), and pathological states, such as infectious diseases and inflammation. ABS is an original hemostatic factor within many junction of hemostasis, neoplasia, and infection. Our findings cast future experimental and clinical ABS research to be placed in the clinical management of *H. pylori*-induced GI lesions.
